# Chromosomal damage, gene expression and alternative transcription in human lymphocytes exposed to mixed ionizing radiation as encountered in space

**DOI:** 10.1038/s41598-024-62313-7

**Published:** 2024-05-20

**Authors:** Milagrosa López Riego, Prabodha Kumar Meher, Beata Brzozowska, Pamela Akuwudike, Martin Bucher, Ursula Oestreicher, Lovisa Lundholm, Andrzej Wojcik

**Affiliations:** 1 Centre for Radiation Protection Research, Department of Molecular Biosciences, The Wenner-Gren Institute, Stockholm University, Svante Arrhenius Väg 20C, 106 91 Stockholm, Sweden; 2 Institute of Biology, Jan Kochanowski University, Kielce, Poland; 3 Institute of Experimental Physics, Faculty of Physics, University of Warsaw, Warsaw, Poland; 4 Federal Office for Radiation Protection, Oberschleissheim, Germany

**Keywords:** Alpha radiation, X-rays, Mixed beams, Space radiation, Chromosomal aberrations, Gene expression, Cancer risk, Astronauts, Gene expression, DNA damage and repair, Biophysics, Cancer, Cell biology, Biomarkers

## Abstract

Astronauts travelling in space will be exposed to mixed beams of particle radiation and photons. Exposure limits that correspond to defined cancer risk are calculated by multiplying absorbed doses by a radiation-type specific quality factor that reflects the biological effectiveness of the particle without considering possible interaction with photons. We have shown previously that alpha radiation and X-rays may interact resulting in synergistic DNA damage responses in human peripheral blood lymphocytes but the level of intra-individual variability was high. In order to assess the variability and validate the synergism, blood from two male donors was drawn at 9 time points during 3 seasons of the year and exposed to 0–2 Gy of X-rays, alpha particles or 1:1 mixture of both (half the dose each). DNA damage response was quantified by chromosomal aberrations and by mRNA levels of 3 radiation-responsive genes *FDXR*, *CDKN1A* and *MDM2* measured 24 h post exposure. The quality of response in terms of differential expression of alternative transcripts was assessed by using two primer pairs per gene. A consistently higher than expected effect of mixed beams was found in both donors for chromosomal aberrations and gene expression with some seasonal variability for the latter. No synergy was detected for alternative transcription.

## Introduction

A major problem associated with space travel are the health effects of ionizing radiation^1^. Astronauts travelling on missions beyond low Earth orbit (LEO) will be primarily exposed to galactic cosmic rays (GCR) and solar particle events (SPE) that are largely protons^2^. While passing through the structure of the spacecraft and astronauts’ bodies, primary particles undergo nuclear interactions, producing a wide variety of secondary particles including neutrons and photons^3^. In effect, the radiation environment inside a spacecraft is different from that encountered in empty space^4^. Major health consequences from exposure to ionizing radiation during space travel are cancer, cardiovascular disease and cognitive impairment^5^.

In order to protect astronauts from unacceptable risk of cancer, the National Aeronautics and Space Administration (NASA) established a space permissible exposure limit according to which the risk of exposure-induced death (REID) due to cancer may not exceed 3%^5^. The dose corresponding to the limit is calculated by multiplying organ specific absorbed doses in units of Gy by a dimensionless, radiation-type specific, quality factor Q that expresses the biological effectiveness of charged particles as a function of their unrestricted linear energy transfer (LET) in water^2^. The product corresponds to the organ dose equivalent in units of Sv. Dose equivalents of exposed organs are then summed yielding a quantity known as the effective dose, also in units of Sv.

Most of the uncertainty in estimating organ dose equivalent values is associated with quantifying the quality factor Q^5^. Relative biological effectiveness values for the full spectrum of GCR and SPE cannot be derived from measurements because the space radiation environment cannot be reproduced on Earth. Hence, Q values are derived from Monte Carlo simulations and different approaches exist to best reflect the biological effectiveness based on LET and the track structure of charged particles^2^. What is not considered in simulations is the possible effect from interaction of high LET charged particles with low LET photons. The flux of photons arising from the interaction of charged particles with the spacecraft and astronauts’ bodies is lower than that of the primary particles but high enough to be of concern from the perspective of health effects^3^. Indeed, the effect of exposure to mixed high- and low-LET space radiation field is discussed by NASA as a source of uncertainty in calculating REID but its magnitude is not known due to lack of data^5^. A further uncertainty is related to potential interactions between the primary charged particles of various types and energies, leading to enhanced biological effectiveness beyond that resulting from summed Q values^6^.

In addition to the radiation field, astronauts in space are exposed to microgravity that is known to have a wide array of negative effects on the human body^7^, including muscle atrophy^8^, that is related to mechanical unloading leading to differential gene expression and alternative splicing^9^. Microgravity-induced alternative splicing is possibly not restricted to muscle cells, as altered regulations of gene expression has been detected in peripheral blood mononuclear cells (PBMC) of astronauts^10^ and in plants shipped to space^11^. Alternative splicing can lead to modified activity of tumor suppressor genes and, consequently, to cancer induction and progression^12^. Gamma radiation also induces alternative splicing^13^. Given the fact that space travel is inevitably associated with exposure to both microgravity and radiation, the precise causative factor for alternative splicing observed in space is difficult to discern.

The present investigation was undertaken with the aim of studying the impact of mixed low- and high-LET radiation exposure on chromosomal aberrations and expression of DNA damage responsive genes and their alternative transcription in PBMC of two human donors. To this end we used a dedicated mixed beam exposure facility^14^ that has been used in a number of studies to demonstrate that low- and high-LET radiations interact leading to synergistic effects^15–21^. We also observed that the synergistic effect seen at the level of gene expression in PBMC is prone to inter- and intraindividual variability, the causes of which are not understood^21^. Individual response to radiation can be regarded as a continuous, polygenic trait^22^. Polygenic traits are strongly influenced by the environment and stochastic molecular variation^23^ so the question arises regarding the validity and significance of a synergistic response observed in a few experiments. In order to address this question, we collected PBMC from two donors on nine occasions, allowing to evaluate the possible seasonal impact on the response. Blood was drawn and exposed to mixed beams during three to five consecutive weeks (intra-seasonal variability) during three seasons of the year (inter-seasonal variability). The focus was on deep analysis of two donors who, in an earlier study, have shown variability in response to mixed beams^24^.

## Results

### Aberration frequencies in PBMC of both donors exposed to mixed beams are consistently higher than expected with the relative biological effectiveness of mixed beams similar to that of alpha particles

**Figure 1:**
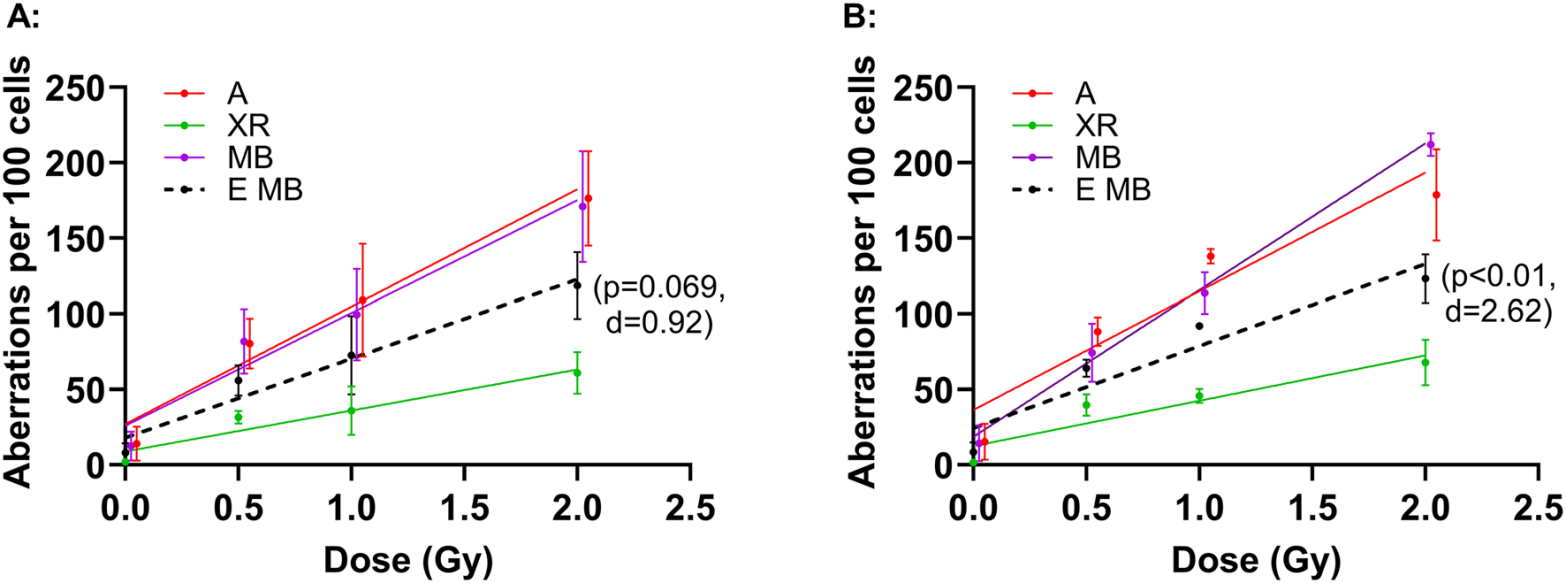
Mean chromosomal aberrations in PBMC from donor 1 (**A**) and donor 2 (**B**) exposed to X-rays (green) and alpha particles (red) and mixed beams (purple). Expected dose responses for mixed beams is depicted as dashed black line. Symbols represent mean results from three seasons and are nudged on the X-axis for clarity. Error bars represent standard deviations. Data were fitted to linear functions, the slopes are given in Supplementary Table 1. T-test p values and Cohen’s effect size d values are shown in brackets for MB versus E MB slope comparisons. *PBMC* peripheral blood mononuclear cells, *A* alpha radiation, *XR* X-rays, *MB* mixed beams, *E MB* expected mixed beams.

To verify if the synergistic effect of mixed photons and alpha particles in PBMC reported earlier^15–21^ is consistent and not the outcome of temporary, seasonal variability, dose response relationships for chromosomal aberrations were analyzed in samples from two donors collected repeatedly 9 times over a time span of 3 seasons. Freshly collected whole blood was exposed to increasing doses of X-rays, alpha particles and a 1:1 mixed beam (MB) of both radiations (half the dose each). Aberrations were scored in Giemsa-stained chromosomes of first post-treatment mitoses. The possible confounding impact of cell cycle delay and resulting underestimation of aberrations in cells exposed to high-LET radiation was avoided by treating cells exposed to all radiation types with calyculin-A^25^ that induces premature chromosomal condensation of cells blocked in the G2 phase of the cell cycle^26^. The aberration frequencies were fitted to a linear function and the slope values were derived (see Supplementary Table 1 for values). When results from repeated experiments were combined, a synergistic effect of X-rays and alpha particles was seen, with aberration frequencies in the mixed beam group higher than expected based on assumed additivity of the single beam components. The effect was somewhat lower in PBMC of donor 1 (large effect size but not significant) than in PBMC of donor 2 (very large effect size and highly significant). Alpha particles were more efficient than X-rays in inducing aberrations (Fig. 1), an expected outcome resulting from the high biological effectiveness of alpha radiation. In order to analyze the consistency of the results, ratios of dose–response slopes from mixed beam and expected groups were plotted for each experiment (Fig. 3A, top rows of ratios designated ABER). For donor 1, the results of 8 out of 9 experiments pointed towards synergism, with a mean ratio of 1.42 and week ratio values between 0.92 and 2.43. Only in week 3 of season 3 was the ratio lower than 1. For donor 2, the mean ratio was 1.95 and week ratio values were between 1.03 and 3.6. Hence, it can be concluded that the consistency of the synergistic effect at the level of chromosomal damage is high.

Using X-rays as the reference radiation, relative biological effectiveness (RBE) values of alpha particles and MB were calculated from the slope values and are shown in Table 1. For both donors pooled, the RBE value of mixed beams was weakly higher (Cohen’s effect size d = 0.33) than that of alpha particles (3.00 ± 0.87 and 2.73 ± 0.78, respectively), a difference that was largely driven by the large size of the effect in PBMC of donor 2 (Cohen’s effect size d = 0.85).

**Table 1:** Relative biological effectiveness (RBE) of alpha particles (alphas) and mixed beams (MB) calculated as the ratio of slopes for X-rays to alphas and X-rays to MB derived from chromosomal aberration analyses. The doses to reach 10 aberrations per 100 cells are shown for illustrative purposes. The mean values and standard deviations (std) were calculated from 9 repeated examinations per donor. Cohen’s effect size d values for difference between RBE values of alphas and MB are: 0.11 (donor 1); 0.85 (donor 2) and 0.33 (donors 1 and 2). The corresponding p-values (Student’s t-test) were < 0.05.

Donor	Radiation type	Slope	Dose (Gy) to reach 10 aberrations	RBE
		Mean ± std	Mean ± std	Mean ± std
1	X-rays	27.16 ± 10.69	0.37 ± 0.14	1 ± 0
	Alphas	77.63 ± 19.85	0.13 ± 0.03	2.86 ± 0.73
	MB	74.57 ± 31.11	0.13 ± 0.06	2.75 ± 1.15
2	X-rays	30.03 ± 10.35	0.33 ± 0.11	1 ± 0
	Alphas	78.51 ± 24.63	0.13 ± 0.04	2.61 ± 0.82
	MB	97.06 ± 18.53	0.10 ± 0.02	3.23 ± 0.62
1 & 2	X-rays	28.60 ± 10.52	0.35 ± 0.13	1 ±
	Alphas	78.07 ± 22.24	0.13 ± 0.04	2.73 ± 0.78
	MB	85.82 ± 24.82	0.12 ± 0.03	3.00 ± 0.87

### mRNA levels of FDXR, CDKN1A and MDM2 alternative transcripts in PBMC of both donors exposed to mixed beams are higher than expected with distinct seasonal variability

**Figure 2:**
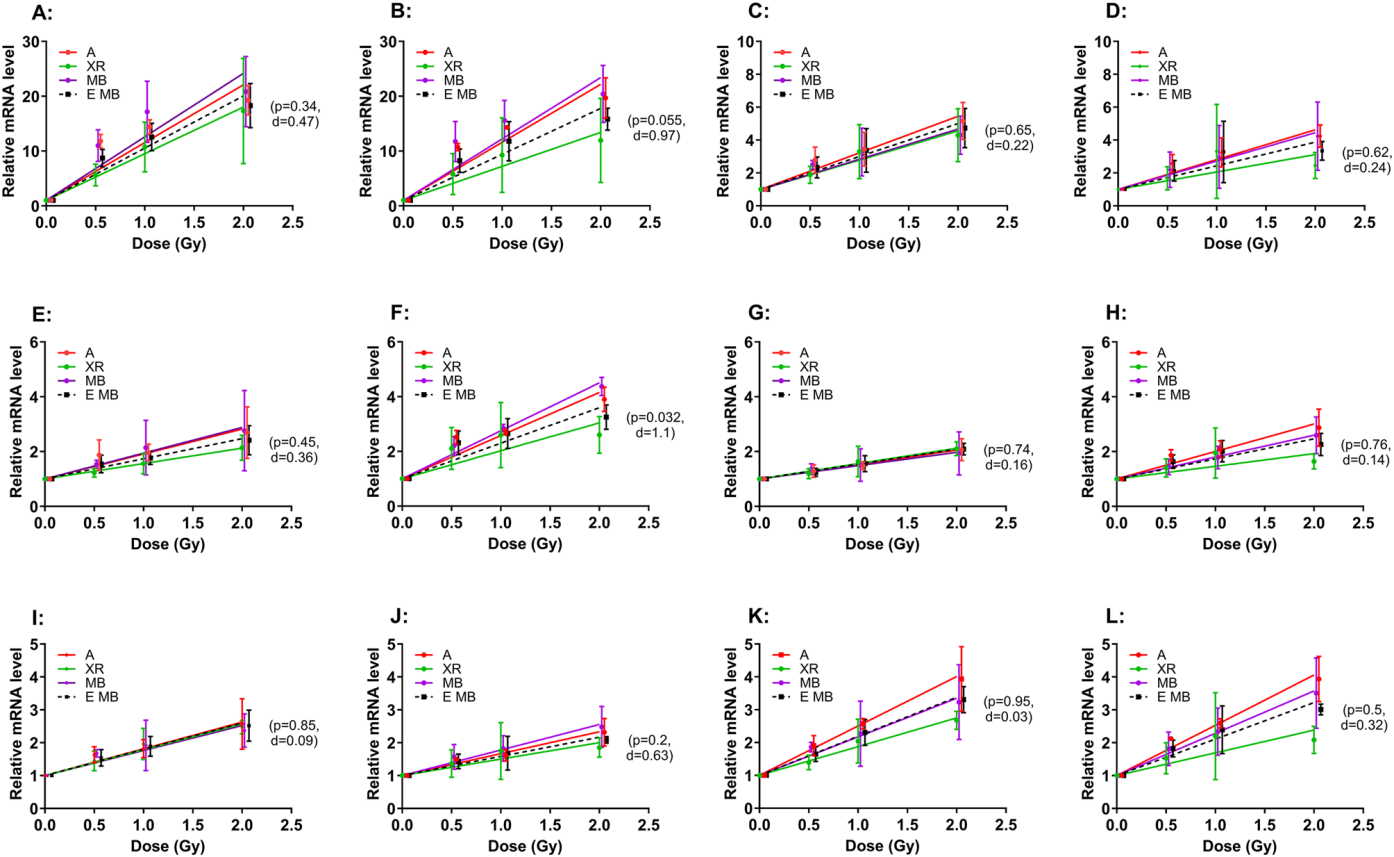
Relative mRNA levels of *FDXR*, *CDKN1A* and *MDM2* alternative transcripts targeted by each primer pair 24 h after exposure to X-rays (XR, green), alpha particles (**A**, red) and mixed beams (MB, purple) in PBL from two donors (donor 1: left panels; donor 2: right panels) obtained in triplicate during 3 seasons. Expected dose response for mixed beam (EMB) is depicted as dashed black lines. Symbols represent mean result from three seasons and are nudged on the X-axis for clarity. Each seasonal mean was calculated from 3 intra-seasonal repeats. Error bars represent standard deviations. Data were fitted to a linear function; slopes are given in Supplementary Table 2. T-test p values and Cohen’s effect size d values are shown in brackets for MB versus EMB slope comparison. PP1 and PP2; V1 and V2; 303/304 and 315 represent primers specific for certain alternative transcripts of the genes FDXR; CDKN1A; and MDM2, respectively. See materials and methods section “gene expression analysis by qRT-PCR” for more details.

Next, it was interesting to see if the consistent synergistic effect of mixed photons and alpha particles observed at the level of chromosomal damage is detected at the mRNA level of the three known radiation-responsive genes *FDXR*, *CDKN1A* and *MDM2*^27^ and if mixed beam exposure leads to atypical alternative transcription. To this end we used RT-qPCR to measure the levels of expression of different alternative transcripts targeted by two different primer pairs per gene, 24 h post exposure, using aliquots of the same blood samples that were used for chromosomal aberration analysis. The mean mRNA expression relative to 0 Gy samples (2^−∆∆Ct^) from all blood collections per donor and primer-pair specific transcript variants are shown in Fig. 2, fitted to linear function. The slope values were derived and are given in the Supplementary Table 2. Similarly, as for chromosomal aberrations, alpha particles induced the highest level of response, exceptions being CDKN1A V4 in donor 1 (panel G) and MDM2 303/304 in donor 1 (panel I) where the slopes of all radiation types were identical. Generally, the observed levels of gene expression induced by mixed beams were higher than expected, but the difference was significant and very large only for CDKN1A V1 in donor 2 (panel F).

**Figure 3:**
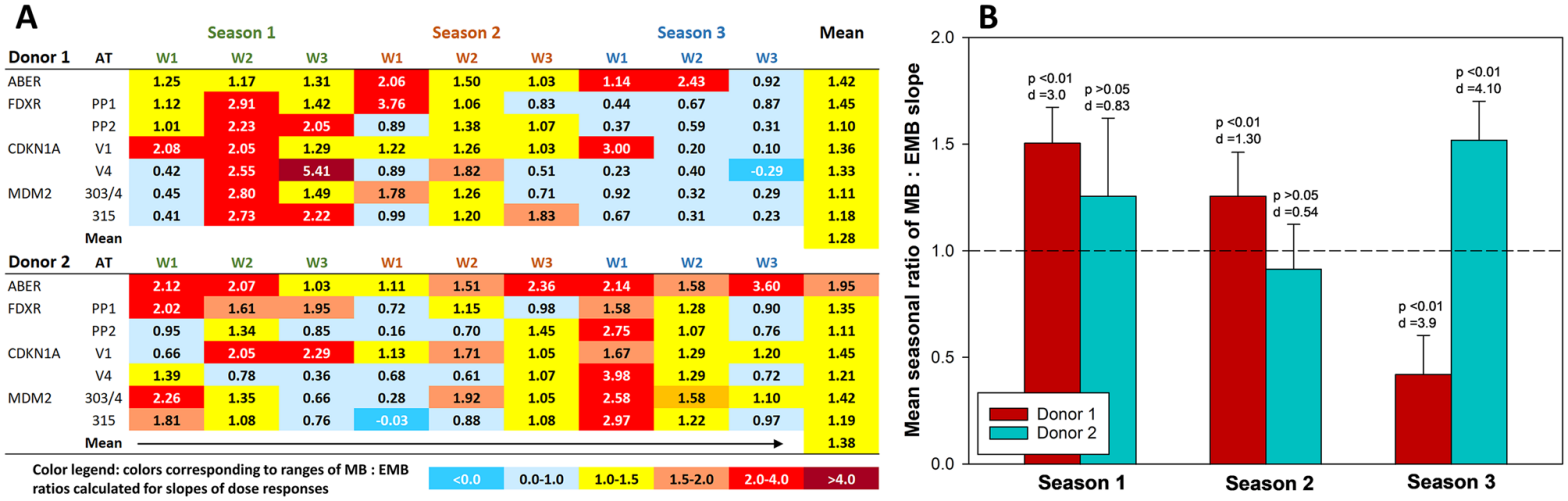
Weekly (**A**) and mean seasonal (**B**) ratios of observed mixed beam (MB) to expected mixed beam (EMB) slope values calculated for gene expression and chromosomal aberrations in PBMC of donors 1 and 2. (**A**) A heat map where colors corresponding to ranges of ratios (selected arbitrarily for optimal representation) are explained in the legend below the panel. ABER: aberrations. *FDXR*, *CDKN1A* and *MDM2*: analyzed genes. PP1, PP2, V1, V4, 303/4, 315: alternative transcript-specific primer pairs. W1–W3: weeks of the seasons. (**B**) Mean seasonal ratios and standard deviations from values of all genes and aberrations. T-test p values and Cohen’s effect size d values are shown for each mean ratio compared to the expected value of 1.

In order to analyze the consistency of the results, ratios of slopes for MB and expected mixed beams (EMB) were calculated for each endpoint and are given in Fig. 3A. Each row of ratios is designated with the gene name and primer pairs transcript variant. Results from analyzes of aberrations are also included (ABER rows). In case of two weekly ratios, the values were negative because negative slopes were obtained for MB (donor 1 CDKN1A V4 in week 3 of season 3 and donor 2 MDM2 315 in week 1 of season 2). For donor 1, a particularly strong synergistic effect was seen during week 2 of season 1 and for donor 2 during week 1 of season 3. With exception of CDKN1A V1, all ratios in donor 1 during season 3 were lower than 1, indicating subadditivity of X-rays and alpha particles. For week 3, this correlated with the ratio derived from chromosomal aberrations. No similarly consistent seasonal effect was seen for donor 2. Despite variabilities in the weekly values, the mean values (Fig. 3A, rightmost column) from all weeks per analyzed endpoint are all higher than 1, suggesting a consistent synergistic effect when all results per endpoint are pooled.

The seasonal differences in mean ratios of each donor are graphically shown in Fig. 3B. For donor 1, the seasonal ratios were significantly and very largely higher than 1 during seasons 1 and 2 and significantly and very largely lower than 1 during season 3. For donor 2, the ratios were non-significantly but largely higher than 1 during season 1 and significantly and very largely higher than 1 during season 3, while the difference during season 2 was somewhat lower but not significantly different from 1.

It can be concluded that the mode of interaction between X-rays and alpha particles is prone to seasonal intra-donor, and inter-donor variation.

### Modification of alternative transcription by radiation is quality-dependent

**Figure 4:**
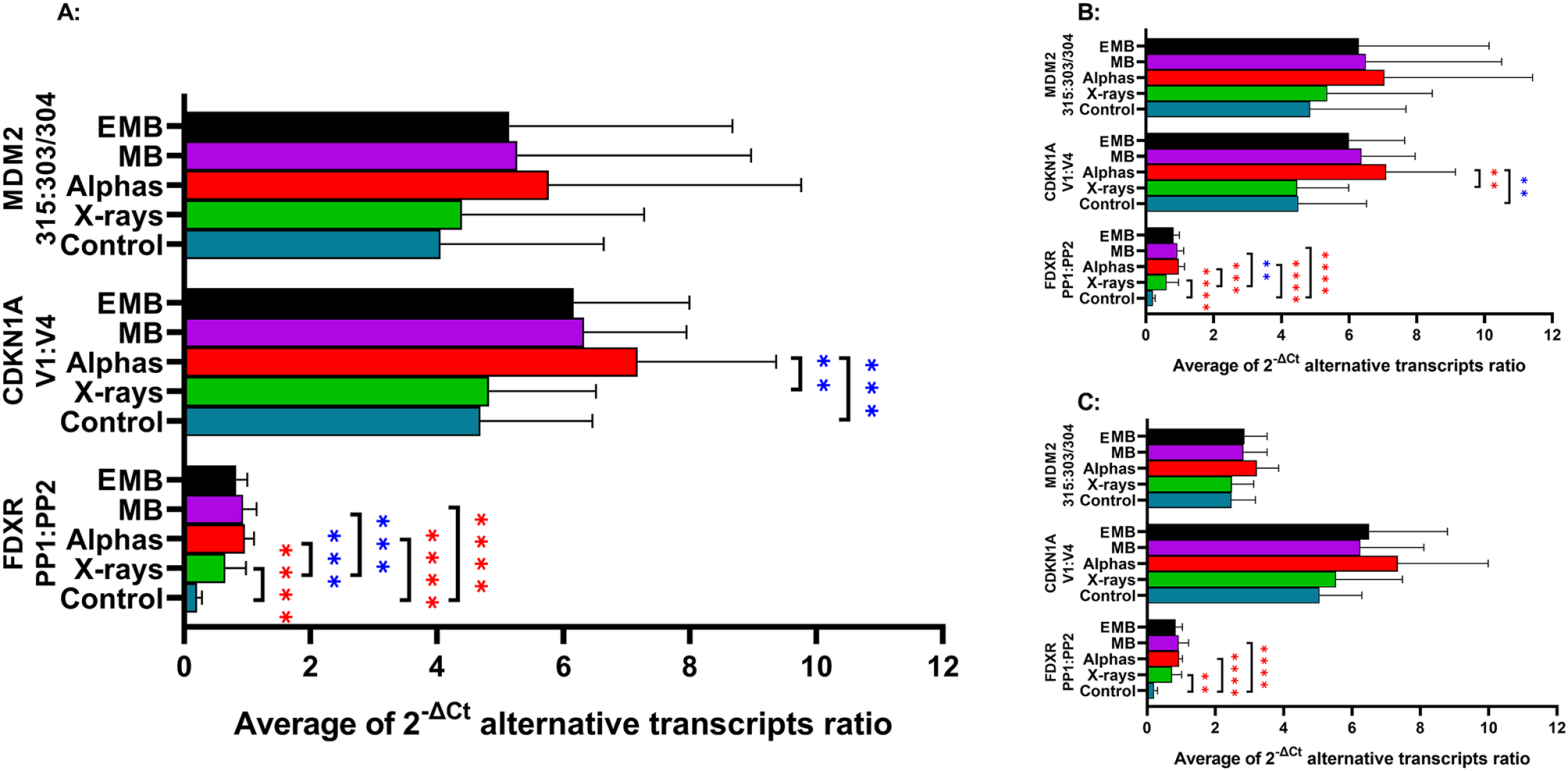
Ratios of the average of 2^−∆Ct^ values observed in mononuclear cells of both donors for each primer pair per gene: *MDM2* (315:303/304), *CDKN1A* (V1:V4), *FDXR* (PP1:PP2). The average is calculated for the primer pair ratios of mean values of irradiated samples (0.5–2 Gy mixed beams observed (MB) or expected (E MB), alpha particles or X-rays) or controls (0 Gy, in triplicate per sampling timepoint) obtained in each sampling timepoint. (**A**) Pooled results of donors 1 and 2 from all seasons. (**B**) Pooled results of donors 1 and 2 from seasons when MB:EMB ratio > 1 (see Fig. 3B), (**C**) Pooled results of donors 1 and 2 from seasons when MB:EMB ratio was ≤ 1 (see Fig. 3B). Error bars represent standard deviations. One-way ANOVA Šídák's multiple comparisons test and Cohen’s effect size results are shown for comparisons which have a significant adjusted p value according to: 0.1234 (non-significant), 0.0332 (*), 0.0021 (**), 0.0002 (***), < 0.0001(****). The color of the asterisks indicate effect size: medium (> 0.5–0.8, green), large (> 0.8–1.3, blue), very large (≥ 1.3, red).

In order to analyze if radiation, and the radiation quality in particular, has an impact on alternative transcription, expression levels of the transcript variants analyzed by each primer pair were compared. To this end, ratios of the net transcript levels normalized to the reference gene (2^−∆Ct^) were calculated as FDXR PP1:PP2, CDKN1A V1:V4 and MDM2 315:303/304 for each week and donor. The results, pooled for both donors and all seasons, are shown in Fig. 4A. For *FDXR*, the ratio of PP1 to PP2 transcripts was significantly and very largely higher in irradiated lymphocytes as compared to control, irrespective of the radiation quality (Supplementary Table 3). The ratio was radiation quality-dependent, as its value following alpha particles and mixed beams was significantly and largely higher than after X-rays. For *CDKN1A*, the ratio of the expression of V1 to V4 transcripts was significantly and largely higher after alpha particle exposure as compared to control, but not so after mixed beams and X-rays. The increase after mixed beams as compared to control was large but not statistically significant. For *MDM2*, the ratio of 315:303/304 was only increased with a medium effect with no statistical difference between radiation qualities (see Supplementary Table 3 for values). Taken together it can be concluded that alternative transcription is radiation quality dependent with alpha particles having a stronger impact than X-rays. The impact of mixed beams is not different from the expected level.

In order to further investigate whether the impact of radiation quality on the level of alternative transcripts was related to the synergistic action of X-rays and alpha particles, the same analysis as above was carried out separately for seasons during which synergy dominated (donor 1 seasons 1 + 2 and donor 2 seasons 1 + 3—see Fig. 3A) and when it did not (donor 1 season 3 and donor 2 season—see Fig. 3A). The results for seasons with synergy are shown in Fig. 4B and for seasons with no synergy in Fig. 4C. For seasons with synergy, the pattern of transcript variant ratios was similar as for all seasons pooled. The same is true for seasons with no synergy, but the differences between the treatments were generally weaker. Nevertheless, both donors showed a very large difference in the ratio of *FDXR* transcripts in seasons with and without synergy. For MDM2 the ratios during seasons without synergy were lower with lower scatter than for seasons with synergy. This could be due to the lower number of analyzes included in the non-synergy group. The low number of analyzes can also explain the lack of significant differences between radiations in CDKN1A. Altogether, the results suggest that alternative transcription is not related to the mode of interaction between X-rays and alpha particles.

## Discussion

The results of this study demonstrate that high- and low-LET radiations interact giving rise to higher than expected levels of cytogenetic damage and gene activation. DNA and chromosomal damage is known to be causally associated with cancer induction^28,29^. Hence, the results indicate that, for mixed beam exposure as encountered in space, cancer risk may be underestimated if it is calculated based on the assumption of simple effect additivity (SEA) between the various mixed beam components. This is the current practice at NASA^5^ and the International Commission on Radiological Protection (ICRP)^30^. For the sake of clarity, it should be explained that NASA considers the radiation quality in estimating the probability of stochastic effects resulting from planned exposures by multiplying the absorbed dose in Gy by a quality factor Q which results in the dose equivalent H_Q_ in Sv^1^. The ICRP uses a radiation weighting factor w_R_ instead of Q and the product of multiplication with the absorbed dose is the equivalent dose H in Sv^30^. While both Q and w_R_ are related to RBE, the w_R_ value is primarily used for practical radiation protection purposes to ensure legal compliance to limits, with simplified values chosen by a committee. ICRP does state that for calculating risks appropriate RBE values should be used instead of w_R_ values^31^. The RBE for chromosomal aberrations of the 1:1 X-rays and alpha particles mixed beam used in the current study was the same as for alpha particles alone and equaled 3. The H_Q_ or H of the mixed beam calculated based on SEA is 2 Sv (0.5 Gy of X-rays × 1 + 0.5 Gy of alpha particles × 3). However, the value derived from the current results shows that the correct way of calculating H is to multiply the total mixed beam dose by the RBE. The difference between the expected H_Q_ or H of 2 Sv and the measured value of 3 Sv demonstrates the magnitude of underestimating the expected levels of stochastic effects by the SEA approach.

Charged particle radiation in space is comprised of high (H) energy protons and high charge (corresponding to an atomic number Z) and energy (E) nuclei (HZE). The large ionization power of HZE ions with Z > 2 makes them a major contributor to the risk, despite of their lower fluence than protons^1^. The alpha particles applied in the current study have a LET close to 100 keV µm^−1^^15^ and, being He nuclei lacking two electrons, have a Z equal to 2. Based on our results, it is impossible to predict the magnitudes of possible synergistic effects between radiations occurring in space. It is interesting to note that approaches are underway to simulate synergies of HZE ions beams. We have checked whether an interaction occurs at the level of cell killing between carbon and oxygen ions but did not find deviations from additivity^32^. With respect to interaction between X-rays and alpha particles, the presented results are in line with the results of our earlier studies with the same mixed beam facility but with various cell models and endpoints^15–21^. It is also worth mentioning that the tracks of HZE particles consist of a high-LET core and low-LET penumbra with delta particles (secondary electrons), which may irradiate structures within a cell further away from the primary track and even surrounding cells. Thus, under conditions of high fluence, HZE particles can themselves produce mixed radiation.

The molecular mechanisms of the interaction are not understood. High- and low-LET radiations differ in the structure of the track along which energy is deposited inside a cell nucleus. Alpha particles induce a highly heterogeneous pattern of DNA damage and breaks along a narrow alpha-particle track resulting in closely spaced, correlated breaks which are more likely to mis-repair and form mutations and aberrations, possibly leading to cell death, compared to the more homogeneously and sparsely distributed breaks produced by X-rays^33^. The complexity of DNA damage increases with LET by virtue of additional strand breaks and/or base damage within a distance of few base pairs^34^. Additionally, mixed beams of low and high LET may potentiate damage complexity at the chromosome level due to mis-repair resulting from pairwise interactions of separate DNA damage sites over large distances. Indeed, we have observed higher than expected frequencies of complex aberrations in PBMC exposed to mixed beams of alpha particles and X-rays^16^. In addition, the mixed beam effect could result from preferential engagement of the DNA repair proteins in removing lesions induced by one type of radiation, so that the lesions resulting from the other type are not properly removed^20,35^. It is also conceivable that the high-LET damage leads to a loosened chromatin conformation, which would make the DNA more susceptible to radicals induced by low-LET radiation^36^. However, considering the maximum irradiation time of ca 30 min and the short half-live of radiation-induced radicals (in the order of nanoseconds^37^), together with evidence on radiation-induced foci (RIF) dynamics using time-lapse florescence microscopy following exposure to different radiation qualities^19,35^, that possibility seems less likely. One other plausible explanation for the mixed beam effect would relate to a lack of mobility of radiation-induced breaks, which could undergo mis-repair in a time scale of hours, in the presence of a high-LET component. Interestingly, the mean square displacement of RIF induced by simultaneous alpha and X-ray exposure, as in our setting, revealed slowest movement of foci induced by the mixed beam^19^. To note, it is reasonable to assume that during the abovementioned maximum irradiation time of 30 min, a fraction of IR-induced DSBs may have already undergone repair, which would translate into a potentially reduced biological effect as compared to more acute exposures. Besides, temperature during irradiation is a known modifying factor of radiation-induced effects, including cytogenetic damage^38^, which may contribute to differences in outcome in studies where mixed beam exposure was conducted at 37 °C, such as^39^. The dose responses of some endpoints applied in the earlier mixed beam studies, such as the comet assay^20^ or 53BP1 foci^18^ were not linear, necessitating the construction of isobolograms and envelopes of additivity to test if the interaction was significantly different from the expected value^40^. In the present investigation, we decided to fit the results to linear functions, making it possible to calculate the expected levels of response by simple arithmetic. Some results, such as chromosomal aberrations after alpha radiation, appear to flatten-off at 2 Gy, making a non-linear fit an option. However, such fitting would not change the conclusions regarding the effect of mixed beams. Moreover, the results are relevant for space travel, where the total doses absorbed by astronauts should not exceed 1 Sv^5^.

In addition to quantitative analysis of DNA damage by chromosomal aberrations and damage response by gene expression, we also looked at a qualitative aspect of the response by analyzing alternative transcription that is a term describing the broader process including both alternative splicing as well as alternative promoter usage. Alternative splicing is known to play a role in the process of carcinogenesis^12^ and is modulated by ionizing radiation^13^. Three genes were selected for analysis based on earlier results that demonstrated their higher than expected upregulation by mixed beams of alpha particles and X-rays^21^. Moreover, these genes are known to be highly responsive to radiation exposure, so their level of expression is used as biomarker of exposure^27^. Two primer pairs per gene were selected due to their ability to target radiation-responsive transcript variants: PP1 and PP2 for the *FDXR*^13^, V1 and V4 for *CDKN1A*^41^ and 303/304 and 315 for *MDM2*^42^. In contrast to the quantitative results, no synergy of X-rays and alpha particles was detected at the level of alternative transcription. Overall, radiation changed the ratio of transcript variants, with alpha particles having a stronger impact than X-rays. The pattern was seen both during seasons with and without synergism, further confirming that alternative splicing is not influenced by interaction of X-rays and alpha particles. The fact that alternative splicing is influenced by radiation and its quality (X-rays vs alpha particles) is relevant for predicting the health effects of space travel. Changes in the expression of the alternative splicing-regulating snoRNA molecules were observed in lymphocytes of astronauts returning from space shuttle missions^10^. How far these changes were induced by microgravity and how much by space radiation is not known and it cannot be excluded that both factors interacted.

Individual response to radiation can be regarded as a continuous, polygenic trait^22^. Polygenic traits result from the interaction of genetic and environmental factors and estimates on the relative contribution of each to the observed phenotype variance requires careful interpretation^43^. In addition, molecular noise contributes to phenotype plasticity^23^ and stochastic variation in gene expression may be a contributing factor^44^. In an earlier study we noticed that the synergistic effect of mixed beams at the level of gene expression in PBMC of two donors showed inter- and intra-individual variability^21^. In order to identify the nature vs nurture source of the variability we selected two individuals from the earlier study (donors 3 and 4, now denoted as 1 and 2, respectively) and analyzed the response in PBMC repeatedly 3 times at weekly intervals during three seasons separated by 3 months. For the synergistic effect of mixed beams we saw a somewhat stronger level of variability at the level of gene expressions as compared to chromosomal aberrations. This is not surprising given that chromosomal aberrations are the outcome of a complex DNA damage response process that is regulated by multiple genes, the expression of which is randomly variable^44^. The impact of variation in expression of individual genes will average out at the level of a complex process that they steer. Interestingly, the level of the synergistic effects in PBMC in donor 1 showed a consistent drop during season 3, suggesting that some seasonal, environmental factors could influence the response. However, the effect was not seen in PBMC of the second donor (whose samples were analyzed in parallel to those of donor 1). Also, donor 1 did not recall any abnormal state of health during season 3. The level of synergism also tended to show weekly patterns (e.g. donor 1 season 1 week 2 and donor 2 season 3 week 1). However, the patters were not consistent between the donors and the majority of ratio values appeared to be randomly variable. Hence, the seasonal or weekly changes in synergism are likely the outcome of chance, although a more solid conclusion would require further repeated analyzes with PBMC of a higher donor number. The present results suffice to conclude that, despite single repeat variability, the synergistic effect of X-rays and alpha particles is consistent in PBMC of both donors.

A note should be added regarding the scoring of chromosomal aberrations. In order to prevent contamination of slides with second division mitoses, colcemid was added to the blood cultures for the last 28 h^45^. Moreover, in order to prevent cell cycle delay of cells heavily damaged by alpha particles^25^, calyculin-A was added to all cultures for the last 1.5 h to chemically induce premature chromosome condensation^26^. Despite promising earlier results with this approach, the quality of the achieved mitoses was insufficient for unequivocal identification of structural chromosomal aberrations such as dicentrics. Therefore, analyzes focused on counting chromosomes and fragments. This approach is similar to the validated method of counting chromosome fragments following fusion-induced premature chromosome condensation^46^. Scoring chromosomal aberrations on Giemsa-stained slides allows only for the detection of unstable-type aberrations. Analysis of stable-type aberrations requires chromosome painting^47^. It is known that high-LET radiation induces aberrations of high complexity, the detection of which requires chromosome painting^47^. We have previously used chromosome painting to study the effect of combining alpha particles and X-rays and observed higher than expected frequencies of complex aberrations in PBMC exposed to mixed beams^16^. Given the large number of mitoses analyzed in the present study (in total 10,800 mitoses: 50 mitoses per dose point, 9 repeats, 4 doses, 3 radiation types, 2 donors) we decided to score unstable-type aberrations on Giemsa-stained chromosomes. There is no doubt that the obtained frequency and complexity of chromosome aberrations is underestimated, especially following alpha particle and mixed beam exposure. Nevertheless, the meaningful dose responses and RBE for alpha particles, and the observed synergism between alpha particles and X-rays that is consistent with the earlier study^16^ demonstrate that the scored aberrations reliably represent the reaction of cells to the studied radiations.

A further aspect worth mentioning is that the study was performed during the COVID-19 pandemic. While the volunteers were without symptoms at the different sampling times, we cannot exclude a potential effect of vaccination on our results nor the possibility of an ongoing infection without symptoms. With respect to the influence of COVID-19 on the DNA damage response at the gene expression level following radiation exposure, it has previously been shown that the expression of p53 target genes, including FDXR, is reduced in PBMC of COVID-19 patients 24 h after X-ray exposure^48^. According to these results, it is therefore possible that the observed responses might be confounded by COVID-19, and that, had the study been conducted prior to the pandemic, the magnitude of the responses would perhaps be different. It also cannot be excluded that this confounder contributed to the observed intraindividual variability.

Limitations of the study include the high dose rate at which the doses of alpha particles and X-rays were applied and the low number of donors, with only male participants. The advantage of high dose rate is that doses that give significant biological responses can be reached in a short time. However, the dose rates encountered in space are much lower than those studied by us. Nevertheless, the results demonstrate that both radiation types can interact although their relevance to the space environment, that is—*nota bene*—composed of a much more complicated mixed radiation field, must yet be established. Despite the low number of donors, the study demonstrates that synergism of X-rays and alpha particles is a real, consistent effect and not the outcome of random response variability. A follow up study including a larger population size, and preferably, with both male and female participants, is highly demanded to best inform space agencies. Radiological protection of astronauts in space must be at the individual level, so this effect should be considered in planning space missions.

## Materials and methods

### Blood collection and irradiation for gene expression and chromosomal aberration analysis

Fresh peripheral blood was drawn in parallel from two healthy male non-smoking donors of similar age (61 and 63) who had shown the strongest *FDXR*, *CDKN1A* and *MDM2* gene expression variability in a previous study (donors 3 and 4)^21^. Both donors were healthy, of low alcohol consumption and body mass index in the healthy weight range. Blood was collected three times once per week within 3–5 consecutive weeks, denoted as one season. This was repeated during three seasons, separated by approximately three months, resulting in 3 × 3 collection time points per donor. Season 1 was in November–December 2020, the season 2 in February 2021 and season 3 in May 2021. Blood was drawn by venipuncture using a vacutainer (BD, USA) and collected in heparin blood collection tubes (VACUTEST kima, Italy). Shortly thereafter it was spread evenly at the center of a 155 mm diameter custom made polyamide (PA) disc (Institute for Energy-JRC, Netherlands), covered with a 2.5 µm thick Mylar foil (Goodfellow, UK) lid, and exposed or sham-exposed. The exposures at increasing doses, i.e. 0, 0.5, 1, and 2 Gy, of X-rays, alpha particles or 1:1 X-rays and alpha particles (half the dose each) for a mixed beam exposure, were performed as described earlier^21^ at room temperature.

The mixed beam irradiation facility and its dosimetry is described in detail in^14,15^. It consists of a movable shelf for the PA disc. X-irradiation (YXLON SMART 200 X-ray tube, 190 kV, 4.0 mA, filtered through 1.5 cm of Al) alone was carried out in the low table position at a dose rate of 0.068 Gy/min. Exposure to alpha particles (^241^Am, 50.0 ± 7.5 MBq, Eckert & Ziegler Isotope Products GmbH, Germany) was in the top table position at a dose rate of 0.223 Gy/min and an average LET of 90.9 ± 8.5 keV μm^−1^^15^. For exposure to mixed beams the table was in top position where the dose rate of X-rays was 0.052 Gy/min^15^. Mixed beam exposure always started with X-ray and alpha irradiation simultaneously and, when half the total dose was achieved with alpha radiation, the shelf was moved to the bottom position until the other half of the total dose was reached with X-rays.

For gene expression analyzes, 250 µL blood was exposed or sham-exposed on the PA disc and transferred to 14 mL culture tubes (VWR, USA) containing 2.5 mL RPMI 1640 medium supplemented with 10% fetal bovine serum (Sigma-Aldrich, Sweden) 40 U/mL penicillin and 40 μg/mL streptomycin (Sigma-Aldrich, Sweden) and incubated at 37 °C for 24 h. For chromosomal aberration analysis, 250 µL of whole blood was irradiated or sham-exposed, and duplicate cultures were set up in 14 mL culture tubes with 100 µL blood and 3 mL RPMI 1640 supplemented with 10% fetal bovine serum, 40 U/mL penicillin and 40 μg/mL streptomycin and 2.4% phytohemagglutinin (PHA-L, Gibco, USA) and kept in the incubator at 37 °C.

### Gene expression analysis by qRT-PCR

After 24 h culture time, RNA was extracted from blood cultures treated with Red Blood Lysis Buffer (Roche, Germany) using the E.Z.N.A. Total RNA Kit I (Omega Bio-tek, USA) following the manufacturer’s instructions and stored at – 80 °C. RNA concentration was measured by Nanodrop and cDNA was synthesized from 100 ng RNA using the High-Capacity cDNA Reverse Transcription Kit (Thermo Fisher Scientific, Lithuania) with random hexamer primers in a reverse transcription reaction with a final volume of 20 µL and the following cycling conditions: 25 °C for 10 min, 37 °C for 120 min and 85 °C for 5 min, followed by a cooling step at 4 °C. 2 µL cDNA, primers (final concentration 900 nM for genes of interest and 200 nM for *18S*), and 5 × HOT FIREPol EvaGreen qPCR SuperMix (Solis Biodyne, Estonia) were mixed in a final volume of 10 µL and real time PCR reactions were performed in duplicate on a LightCycler 480, starting at 95 °C for 2 min, followed by 45 cycles of 95 °C for 15 s, 60 °C for 20 s and 72 °C for 20 s, plus a final melting cycle at 95 °C for 5 s, 65 °C for 1 min, and continuous 97 °C followed by a cooling step. Non template controls were included in each plate, confirming the absence of contamination. The 2^−ΔΔCt^ method was used for calculation of the relative gene expression and primer specificity was verified by melting curve analysis. Additionally, 2^−∆Ct^ values were used to determine the relative expression of transcripts targeted by each primer pair per gene.

Following up the study by^21^, the three genes that showed the strongest variability, i.e. *FDXR*, *CDKN1A*, and *MDM2*, were selected to analyze the levels of primer-specific alternative transcript variants using two previously described primer pairs per gene, with the naming kept from earlier reports. Primers used were (5′–3′): FDXR PP1 Forward; GCAAGTGGCCTTCACCATTAAG; FDXR PP1 Reverse: CCTTGATCTTGTCCTGGAGACC; FDXR PP2 Forward: GCTTCTGCCACCATTTCTCC; FDXR PP2 Reverse: CTTTAGCAGGTGTTGGGCC^13^; CDKN1A V1 Forward: AGGCACTCAGAGGAGGCGCCA; CDKN1A V1 Reverse: GGTGACAAAGTCGAAGTTCCA; CDKN1A V4 Forward: TGTTTCTGCGGCAGGCGCCAT; CDKN1A V4 Reverse: CCGCCATTAGCGCATCACAGT^41^; MDM2 303/304 Forward: ACCGAGATCCTGCTGCTTT; MDM2 303/304 Reverse: CTCGGGGATCATTCCACTCT; MDM2 315 Forward: TGGCCAGTATATTATGACTAAACGA; MDM2 315 Reverse CACGCCAAACAAATCTCCTA^42^. Genes of interest were normalized to the reference gene *18S* using previously described primers^49^. 18S_Forward: GCTTAATTTGACTCAACACGGGA; 18S_Reverse: AGCTATCAATCTGTCAATCCTGTCC.

### Chromosomal aberration analysis

After 24 h of culture time, 40 µL colcemid (Gibco, USA) was added to the cultures and left until harvest. Calyculin A was added for the last 1.5 h before harvesting at a total culture time of 52 h. Cells were harvested by standard cytogenetic procedure and fixed cells stored at – 20 °C in 800 µL fixative. Fixed cells were shipped to the Federal Office for Radiation Protection, BfS (Oberschleissheim, Germany), where they were dropped on microscopic slides and stained with 8% Giemsa's azur eosin methylene blue solution (Merck KGaA, Germany)/PBS for 5 min. Image acquisition was performed by microscopy using a Metafer Scanning System and Metafer4 Software (MetaSystems Hard & Software GmbH, Germany). A 90% area of prepared slides was scanned at 10 × magnification and metaphases were automatically identified by the metaphase finding module (MSearch) of the Metafer4 software (sensitivity 6.0). The detected metaphases were automatically captured by the autocapture module (Autocapt) on the Metafer4 software at 63 × magnification. High resolution images of mitoses were saved and send back to the Stockholm University for manual scoring. The quality of the chromosome spreads did not allow unequivocal analysis of structural aberrations such as dicentric chromosomes. Therefore, the total number of chromosomes and fragments per metaphase plate was counted. Prior to scoring, the images were coded and scored blind by a single scorer. 50 mitoses per dose point were analyzed resulting in the total number of 450 mitoses per dose point and donor.

### Statistical analysis

Gene expression dose responses were fitted to linear regression Y = slope × X + 1.000, where slopes were forced through 1 considering that data had been normalized to the control, i.e. relative mRNA levels to control or fold change. For chromosomal aberrations, data were fitted to linear regression Y = slope × X + intercept, as different basal level of chromosomal fragments were scored for the different controls and data had not been normalized. The fitting and 2-way ANOVA Šidák multiple slope comparisons tests were performed using GraphPad Prism version 9.4.1. Cohen´s effect size analyzes were performed based on the average and standard deviation of the slopes extracted from the linear regression analyzes. Expected results were calculated by adding the results from single dose components contributing to the mixed beam dose.

### Ethical approval for the experiments

Informed consent was obtained from both donors prior to the experiments. Blood collection, data and sample handling was carried out in accordance with guidelines from the Swedish Ethical Review Authority (Etikprövningsmyndigheten) and the Declaration of Helsinki. The permit number of the Swedish Ethical Review Authority is 2019-03844. Samples were used up during the analysis and no material was stored in a biobank.

### Supplementary Information

Supplementary Information.

## Data Availability

Data supporting the results reported in the article are available upon request from the corresponding author.
