# Supplementary material for: Chromosomal damage, gene expression and alternative transcription in human lymphocytes exposed to mixed ionizing radiation as encountered in space
Source: Sci Rep. 2024 May 20;14:11502. doi: 10.1038/s41598-024-62313-7 (PMC11106305; doi:10.1038/s41598-024-62313-7)
Supplement: Supplementary file 1 — Supplementary Information. [file 41598_2024_62313_MOESM1_ESM.docx]

**Supplemental tables**

Supplemental table 1. Slopes of chromosomal aberration dose responses shown in figure 1. Given are α parameters of fits for results from each week and season. The parameters come from linear fits to the equation Y = αX+β, where Y is the aberration yield and X is the dose. Included are also the mean and standard deviation values calculated for each radiation type per donor. MB: mixed beams, EMB: expected mixed beams, std: standard deviation. EMB slopes were calculated as the mean of respective X-ray and Alpha slopes.

|  | **Radiation** |  | **Week** | | |  |  | **Equation with** |
| --- | --- | --- | --- | --- | --- | --- | --- | --- |
| **Donor** | **type** | **Season** | **1** | **2** | **3** | **mean** | **std** | **fit parameters** |
| **1** | **X-rays** | **1** | 17.14 | 17.94 | 39.66 | 27.16 | 10.69 | Y = 27.16*X + 8.844 |
|  |  | **2** | 37.94 | 24.11 | 42.74 |  |  |  |
|  |  | **3** | 17.49 | 30.29 | 17.14 |  |  |  |
|  |  |  |  |  |  |  |  |  |
| **1** | **Alpha** | **1** | 42.86 | 75.89 | 90.74 | 77.63 | 19.85 | Y = 77.63*X + 27.02 |
|  |  | **2** | 75.43 | 89.26 | 114.9 |  |  |  |
|  |  | **3** | 64.91 | 69.14 | 75.54 |  |  |  |
|  |  |  |  |  |  |  |  |  |
| **1** | **MB** | **1** | 37.60 | 54.74 | 85.37 | 74.57 | 31.11 | Y = 74.58*X + 25.91 |
|  |  | **2** | 117.0 | 84.80 | 81.03 |  |  |  |
|  |  | **3** | 46.86 | 120.90 | 42.86 |  |  |  |
|  |  |  |  |  |  |  |  |  |
| **1** | **EMB** | **1** | 30.0 | 46.92 | 65.2 | 52.4 | 14.14 | Y = 52.39*X + 17.93 |
|  |  | **2** | 56.69 | 56.69 | 78.82 |  |  |  |
|  |  | **3** | 41.20 | 49.72 | 46.34 |  |  |  |
|  |  |  |  |  |  |  |  |  |
| **2** | **X-rays** | **1** | 32.80 | 19.77 | 21.83 | 30.03 | 10.35 | Y = 30.03*X + 12.44 |
|  |  | **2** | 49.14 | 37.14 | 32.69 |  |  |  |
|  |  | **3** | 26.86 | 34.86 | 15.20 |  |  |  |
|  |  |  |  |  |  |  |  |  |
| **2** | **Alpha** | **1** | 72.80 | 102.7 | 123.7 | 78.51 | 24.63 | Y = 78.51*X + 36.36 |
|  |  | **2** | 92.0 | 74.86 | 67.66 |  |  |  |
|  |  | **3** | 55.43 | 75.54 | 41.94 |  |  |  |
|  |  |  |  |  |  |  |  |  |
| **2** | **MB** | **1** | 112.0 | 126.7 | 75.31 | 97.06 | 18.53 | Y = 97.06*X + 18.60 |
|  |  | **2** | 78.40 | 84.46 | 118.40 |  |  |  |
|  |  | **3** | 88.17 | 87.20 | 102.90 |  |  |  |
|  |  |  |  |  |  |  |  |  |
| **2** | **EMB** | **1** | 52.80 | 61.24 | 72.77 | 54.27 | 13.75 | Y = 54.27*X + 24.40 |
|  |  | **2** | 70.57 | 56.00 | 50.18 |  |  |  |
|  |  | **3** | 41.15 | 55.2 | 28.57 |  |  |  |

Supplemental table 2. Slopes of gene and splice variant expression dose responses shown in figure 2. Given are α parameters of fits for results from each week and season. The parameters come from linear fits to the equation Y = αX+β, where Y is the aberration yield and X is the dose. Included are also the mean and standard deviation (std) values calculated for each radiation type per donor. MB: mixed beams, EMB: expected mixed beams. EMB slopes were calculated as the mean of respective X-ray and Alpha slopes.

|  | **Gene** | **Radiation** |  | **Week** | | |  |  | **Equation with** |
| --- | --- | --- | --- | --- | --- | --- | --- | --- | --- |
| **Donor** | **Variant** | **type** | **Season** | **1** | **2** | **3** | **mean** | **std** | **fit parameters** |
| **1** | **FDXR** | **Xrays** | **1** | 1.85 | 2.70 | 9.22 | 8.51 | 5.01 | Y = 8.51*X + 1 |
|  | **PP1** |  | **2** | 5.05 | 7.09 | 9.63 |  |  |  |
|  |  |  | **3** | 17.4 | 10.15 | 13.52 |  |  |  |
|  |  |  |  |  |  |  |  |  |  |
|  |  | **Alpha** | **1** | 15.90 | 7.72 | 10.41 | 10.54 | 2.93 | Y = 10.54*X + 1 |
|  |  |  | **2** | 7.96 | 12.10 | 10.68 |  |  |  |
|  |  |  | **3** | 12.79 | 11.00 | 6.32 |  |  |  |
|  |  |  |  |  |  |  |  |  |  |
|  |  | **MB** | **1** | 9.90 | 15.14 | 13.94 | 11.59 | 5.62 | Y = 11.59*X + 1 |
|  |  |  | **2** | 24.47 | 10.14 | 8.43 |  |  |  |
|  |  |  | **3** | 6.57 | 7.13 | 8.59 |  |  |  |
|  |  |  |  |  |  |  |  |  |  |
|  |  | **EMB** | **1** | 8.88 | 5.21 | 9.82 | 9.53 | 2.76 | Y = 9.53*X + 1 |
|  |  |  | **2** | 6.51 | 9.60 | 10.16 |  |  |  |
|  |  |  | **3** | 15.1 | 10.58 | 9.92 |  |  |  |
|  |  |  |  |  |  |  |  |  |  |
| **2** | **FDXR** | **Xrays** | **1** | 1.92 | 1.50 | 1.96 | 6.2 | 4.11 | Y = 6.2*X + 1 |
|  | **PP1** |  | **2** | 4.62 | 6.02 | 7.01 |  |  |  |
|  |  |  | **3** | 10.47 | 9.33 | 12.96 |  |  |  |
|  |  |  |  |  |  |  |  |  |  |
|  |  | **Alpha** | **1** | 11.86 | 10.75 | 13.17 | 10.58 | 2.28 | Y = 10.58*X + 1 |
|  |  |  | **2** | 9.00 | 11.69 | 10.79 |  |  |  |
|  |  |  | **3** | 10.54 | 12.09 | 5.36 |  |  |  |
|  |  |  |  |  |  |  |  |  |  |
|  |  | **MB** | **1** | 13.91 | 9.88 | 14.74 | 11.2 | 3.74 | Y = 11.2*X + 1 |
|  |  |  | **2** | 4.92 | 10.18 | 8.74 |  |  |  |
|  |  |  | **3** | 16.56 | 13.66 | 8.21 |  |  |  |
|  |  |  |  |  |  |  |  |  |  |
|  |  | **EMB** | **1** | 6.89 | 6.13 | 7.57 | 8.39 | 1.64 | Y = 8.39*X + 1 |
|  |  |  | **2** | 6.81 | 8.86 | 8.90 |  |  |  |
|  |  |  | **3** | 10.51 | 10.71 | 9.16 |  |  |  |
|  |  |  |  |  |  |  |  |  |  |
| **1** | **FDXR** | **Xrays** | **1** | 1.23 | 0.86 | 0.86 | 1.78 | 1.11 | Y = 1.78*X + 1 |
|  | **PP2** |  | **2** | 1.29 | 1.41 | 1.77 |  |  |  |
|  |  |  | **3** | 4.40 | 1.61 | 2.57 |  |  |  |
|  |  |  |  |  |  |  |  |  |  |
|  |  | **Alpha** | **1** | 3.47 | 1.05 | 2.09 | 2.22 | 0.96 | Y = 2.22*X + 1 |
|  |  |  | **2** | 1.22 | 2.62 | 0.95 |  |  |  |
|  |  |  | **3** | 2.52 | 3.32 | 2.76 |  |  |  |
|  |  |  |  |  |  |  |  |  |  |
|  |  | **MB** | **1** | 2.38 | 2.13 | 3.03 | 1.83 | 0.78 | Y = 1.83*X + 1 |
|  |  |  | **2** | 1.12 | 2.79 | 1.45 |  |  |  |
|  |  |  | **3** | 1.28 | 1.46 | 0.82 |  |  |  |
|  |  |  |  |  |  |  |  |  |  |
|  |  | **EMB** | **1** | 2.35 | 0.96 | 1.48 | 2,00 | 0.81 | Y = 2*X + 1 |
|  |  |  | **2** | 1.26 | 2.02 | 1.36 |  |  |  |
|  |  |  | **3** | 3.46 | 2.47 | 2.67 |  |  |  |
|  |  |  |  |  |  |  |  |  |  |
| **2** | **FDXR** | **Xrays** | **1** | 0.65 | 0.63 | 0.43 | 1.06 | 1.08 | Y = 1.06*X + 1 |
|  | **PP2** |  | **2** | 0.43 | 0.41 | 0.64 |  |  |  |
|  |  |  | **3** | 1.05 | 1.50 | 3.78 |  |  |  |
|  |  |  |  |  |  |  |  |  |  |
|  |  | **Alpha** | **1** | 2.04 | 1.78 | 1.84 | 1.81 | 0.62 | Y = 1.81*X + 1 |
|  |  |  | **2** | 1.06 | 2.23 | 1.01 |  |  |  |
|  |  |  | **3** | 3.06 | 1.75 | 1.54 |  |  |  |
|  |  |  |  |  |  |  |  |  |  |
|  |  | **MB** | **1** | 1.28 | 1.61 | 0.96 | 1.72 | 1.57 | Y = 1.72*X + 1 |
|  |  |  | **2** | 0.12 | 0.93 | 1.20 |  |  |  |
|  |  |  | **3** | 5.65 | 1.74 | 2.02 |  |  |  |
|  |  |  |  |  |  |  |  |  |  |
|  |  | **EMB** | **1** | 1.35 | 1.21 | 1.14 | 1.44 | 0.61 | Y = 1.44*X + 1 |
|  |  |  | **2** | 0.75 | 1.32 | 0.83 |  |  |  |
|  |  |  | **3** | 2.06 | 1.63 | 2.66 |  |  |  |
|  |  |  |  |  |  |  |  |  |  |
|  | **Gene** | **Radiation** |  | **Week** | | |  |  | **Equation with** |
| **Donor** | **Variant** | **type** | **Season** | **1** | **2** | **3** | **mean** | **std** | **fit parameters** |
| **1** | **CDKN1A** | **Xrays** | **1** | 0.26 | 1.12 | 0.37 | 0.56 | 0.4 | Y = 0.56*X + 1 |
|  | **V1** |  | **2** | 1.27 | 0.31 | 0.80 |  |  |  |
|  |  |  | **3** | 0.16 | 0.28 | 0.48 |  |  |  |
|  |  |  |  |  |  |  |  |  |  |
|  |  | **Alpha** | **1** | 0.92 | 1.18 | 2.22 | 0.91 | 0.6 | Y = 0.91*X + 1 |
|  |  |  | **2** | 0.31 | 1.06 | 0.4 |  |  |  |
|  |  |  | **3** | 0.22 | 1.03 | 0.87 |  |  |  |
|  |  |  |  |  |  |  |  |  |  |
|  |  | **MB** | **1** | 1.23 | 2.36 | 1.67 | 0.94 | 0.73 | Y = 0.94*X + 1 |
|  |  |  | **2** | 0.96 | 0.86 | 0.62 |  |  |  |
|  |  |  | **3** | 0.57 | 0.13 | 0.07 |  |  |  |
|  |  |  |  |  |  |  |  |  |  |
|  |  | **EMB** | **1** | 0.59 | 1.15 | 1.30 | 0.74 | 0.32 | Y = 0.74*X + 1 |
|  |  |  | **2** | 0.79 | 0.69 | 0.60 |  |  |  |
|  |  |  | **3** | 0.19 | 0.66 | 0.68 |  |  |  |
|  |  |  |  |  |  |  |  |  |  |
| **2** | **CDKN1A** | **Xrays** | **1** | 0.66 | 0.81 | 0.09 | 1.02 | 0.63 | Y = 1.02*X + 1 |
|  | **V1** |  | **2** | 1.43 | 0.29 | 1.92 |  |  |  |
|  |  |  | **3** | 1.76 | 1.00 | 1.24 |  |  |  |
|  |  |  |  |  |  |  |  |  |  |
|  |  | **Alpha** | **1** | 2.42 | 1.24 | 1.24 | 1.58 | 0.4 | Y = 1.58*X + 1 |
|  |  |  | **2** | 2.01 | 1.71 | 1.52 |  |  |  |
|  |  |  | **3** | 1.37 | 1.31 | 1.38 |  |  |  |
|  |  |  |  |  |  |  |  |  |  |
|  |  | **MB** | **1** | 1.01 | 2.10 | 1.52 | 1.75 | 0.45 | Y = 1.75*X + 1 |
|  |  |  | **2** | 1.95 | 1.71 | 1.81 |  |  |  |
|  |  |  | **3** | 2.62 | 1.49 | 1.57 |  |  |  |
|  |  |  |  |  |  |  |  |  |  |
|  |  | **EMB** | **1** | 1.54 | 1.03 | 0.67 | 1.3 | 0.37 | Y = 1.3*X + 1 |
|  |  |  | **2** | 1.72 | 1,00 | 1.72 |  |  |  |
|  |  |  | **3** | 1.57 | 1.16 | 1.31 |  |  |  |
|  |  |  |  |  |  |  |  |  |  |
| **1** | **CDKN1A** | **Xrays** | **1** | 0.97 | 0.57 | -0.05 | 0.56 | 0.37 | Y = 0.56*X + 1 |
|  | **V4** |  | **2** | 0.58 | 0.53 | 1.18 |  |  |  |
|  |  |  | **3** | 0.63 | 0.21 | 0.42 |  |  |  |
|  |  |  |  |  |  |  |  |  |  |
|  |  | **Alpha** | **1** | 1.37 | 0.09 | 0.46 | 0.53 | 0.4 | Y = 0.53*X + 1 |
|  |  |  | **2** | 0.12 | 0.68 | 0.26 |  |  |  |
|  |  |  | **3** | 0.32 | 0.75 | 0.68 |  |  |  |
|  |  |  |  |  |  |  |  |  |  |
|  |  | **MB** | **1** | 0.49 | 0.84 | 1.11 | 0.48 | 0.44 | Y = 0.49*X + 1 |
|  |  |  | **2** | 0.31 | 1.10 | 0.37 |  |  |  |
|  |  |  | **3** | 0.11 | 0.19 | -0.16 |  |  |  |
|  |  |  |  |  |  |  |  |  |  |
|  |  | **EMB** | **1** | 1.17 | 0.33 | 0.21 | 0.54 | 0.28 | Y = 0.54*X + 1 |
|  |  |  | **2** | 0.35 | 0.61 | 0.72 |  |  |  |
|  |  |  | **3** | 0.48 | 0.48 | 0.55 |  |  |  |
|  |  |  |  |  |  |  |  |  |  |
| **2** | **CDKN1A** | **Xrays** | **1** | 0.14 | 0.43 | 0.15 | 0.46 | 0.31 | Y = 0.46*X + 1 |
|  | **V4** |  | **2** | 0.52 | 0.31 | 0.61 |  |  |  |
|  |  |  | **3** | 0.33 | 0.50 | 1.16 |  |  |  |
|  |  |  |  |  |  |  |  |  |  |
|  |  | **Alpha** | **1** | 0.88 | 1.24 | 1.06 | 1,00 | 0.45 | Y = 1*X + 1 |
|  |  |  | **2** | 2,00 | 1.13 | 0.70 |  |  |  |
|  |  |  | **3** | 0.84 | 0.40 | 0.78 |  |  |  |
|  |  |  |  |  |  |  |  |  |  |
|  |  | **MB** | **1** | 0.71 | 0.65 | 0.22 | 0.8 | 0.6 | Y = 0.8*X + 1 |
|  |  |  | **2** | 0.86 | 0.44 | 0.70 |  |  |  |
|  |  |  | **3** | 2.33 | 0.58 | 0.70 |  |  |  |
|  |  |  |  |  |  |  |  |  |  |
|  |  | **EMB** | **1** | 0.51 | 0.84 | 0.61 | 0.73 | 0.25 | Y = 0.73*X + 1 |
|  |  |  | **2** | 1.26 | 0.72 | 0.66 |  |  |  |
|  |  |  | **3** | 0.59 | 0.45 | 0.97 |  |  |  |
|  |  |  |  |  |  |  |  |  |  |
|  | **Gene** | **Radiation** |  | **Week** | | |  |  | **Equation with** |
| **Donor** | **Variant** | **type** | **Season** | **1** | **2** | **3** | **mean** | **std** | **fit parameters** |
| **1** | **MDM2** | **Xrays** | **1** | 1.01 | 0.51 | 0.48 | 0.79 | 0.26 | Y = 0.79*X + 1 |
|  | **303/** |  | **2** | 0.61 | 0.69 | 0.87 |  |  |  |
|  | **304** |  | **3** | 1.11 | 0.64 | 1.16 |  |  |  |
|  |  |  |  |  |  |  |  |  |  |
|  |  | **Alpha** | **1** | 0.81 | 0.36 | 1.87 | 0.81 | 0.54 | Y = 0.81*X + 1 |
|  |  |  | **2** | 0.20 | 0.74 | 0.26 |  |  |  |
|  |  |  | **3** | 0.71 | 1.07 | 1.3 |  |  |  |
|  |  |  |  |  |  |  |  |  |  |
|  |  | **MB** | **1** | 0.41 | 1.22 | 1.75 | 0.76 | 0.48 | Y = 0.76*X + 1 |
|  |  |  | **2** | 0.72 | 0.90 | 0.40 |  |  |  |
|  |  |  | **3** | 0.84 | 0.27 | 0.36 |  |  |  |
|  |  |  |  |  |  |  |  |  |  |
|  |  | **EMB** | **1** | 0.91 | 0.44 | 1.18 | 0.8 | 0.3 | Y = 0.8*X + 1 |
|  |  |  | **2** | 0.41 | 0.72 | 0.57 |  |  |  |
|  |  |  | **3** | 0.91 | 0.86 | 1.23 |  |  |  |
|  |  |  |  |  |  |  |  |  |  |
| **2** | **MDM2** | **Xrays** | **1** | 0.28 | 0.14 | 0.2 | 0.5 | 0.37 | Y = 0.5*X + 1 |
|  | **303/** |  | **2** | 0.33 | 0.13 | 1.06 |  |  |  |
|  | **304** |  | **3** | 0.55 | 0.78 | 1.04 |  |  |  |
|  |  |  |  |  |  |  |  |  |  |
|  |  | **Alpha** | **1** | 0.48 | 0.57 | 1.4 | 0.67 | 0.31 | Y = 0.67*X + 1 |
|  |  |  | **2** | 0.45 | 0.60 | 0.48 |  |  |  |
|  |  |  | **3** | 0.45 | 0.70 | 0.87 |  |  |  |
|  |  |  |  |  |  |  |  |  |  |
|  |  | **MB** | **1** | 0.86 | 0.48 | 0.53 | 0.78 | 0.37 | Y = 0.78*X + 1 |
|  |  |  | **2** | 0.11 | 0.70 | 0.81 |  |  |  |
|  |  |  | **3** | 1.29 | 1.17 | 1.05 |  |  |  |
|  |  |  |  |  |  |  |  |  |  |
|  |  | **EMB** | **1** | 0.38 | 0.36 | 0.80 | 0.58 | 0.23 | Y = 0.58*X + 1 |
|  |  |  | **2** | 0.39 | 0.37 | 0.77 |  |  |  |
|  |  |  | **3** | 0.50 | 0.74 | 0.96 |  |  |  |
|  |  |  |  |  |  |  |  |  |  |
| **1** | **MDM2** | **Xrays** | **1** | 0.96 | 0.62 | 0.23 | 0.88 | 0.35 | Y = 0.88*X + 1 |
|  | **315** |  | **2** | 0.97 | 0.8 | 1.01 |  |  |  |
|  |  |  | **3** | 1.39 | 0.64 | 1.27 |  |  |  |
|  |  |  |  |  |  |  |  |  |  |
|  |  | **Alpha** | **1** | 3.12 | 0.67 | 1.89 | 1.51 | 0.75 | Y = 1.51*X + 1 |
|  |  |  | **2** | 0.95 | 1.69 | 0.73 |  |  |  |
|  |  |  | **3** | 1.25 | 1.73 | 1.52 |  |  |  |
|  |  |  |  |  |  |  |  |  |  |
|  |  | **MB** | **1** | 0.84 | 1.76 | 2.35 | 1.17 | 0.67 | Y = 1.17*X + 1 |
|  |  |  | **2** | 0.95 | 1.50 | 1.59 |  |  |  |
|  |  |  | **3** | 0.88 | 0.37 | 0.32 |  |  |  |
|  |  |  |  |  |  |  |  |  |  |
|  |  | **EMB** | **1** | 2.04 | 0.65 | 1.06 | 1.19 | 0.4 | Y = 1.19*X + 1 |
|  |  |  | **2** | 0.96 | 1.25 | 0.87 |  |  |  |
|  |  |  | **3** | 1.32 | 1.19 | 1.40 |  |  |  |
|  |  |  |  |  |  |  |  |  |  |
| **2** | **MDM2** | **Xrays** | **1** | 0.46 | 0.23 | 0.23 | 0.69 | 0.51 | Y = 0.69*X + 1 |
|  | **315** |  | **2** | 0.57 | 0.20 | 1.17 |  |  |  |
|  |  |  | **3** | 0.56 | 1.20 | 1.61 |  |  |  |
|  |  |  |  |  |  |  |  |  |  |
|  |  | **Alpha** | **1** | 1.37 | 1.71 | 2.26 | 1.53 | 0.32 | Y = 1.53*X + 1 |
|  |  |  | **2** | 1.31 | 1.73 | 1.40 |  |  |  |
|  |  |  | **3** | 1.29 | 1.35 | 1.33 |  |  |  |
|  |  |  |  |  |  |  |  |  |  |
|  |  | **MB** | **1** | 1.66 | 1.05 | 0.95 | 1.29 | 0.75 | Y = 1.29*X + 1 |
|  |  |  | **2** | -0.03 | 0.85 | 1.39 |  |  |  |
|  |  |  | **3** | 2.75 | 1.55 | 1.42 |  |  |  |
|  |  |  |  |  |  |  |  |  |  |
|  |  | **EMB** | **1** | 0.92 | 0.97 | 1.25 | 1.11 | 0.21 | Y = 1.11*X + 1 |
|  |  |  | **2** | 0.94 | 0.97 | 1.29 |  |  |  |
|  |  |  | **3** | 0.93 | 1.28 | 1.47 |  |  |  |

Supplemental table 3. Outcomes of one-way ANOVA Šídák's multiple comparisons test (column Significance) and Cohen´s effect size (column Size) results shown in figure 4. MB: mixed beams, EMB: expected mixed beams.

|  |  |  | **Significance** | **Size** | **Marked on** |
| --- | --- | --- | --- | --- | --- |
| **Figure** | **Gene** | **Comparison** | **p value** | **d value** | **figure as** |
| **4A** | *FDXR* | Control FDXR PP1:PP2 vs. X-rays FDXR PP1:PP2 | <0.0001 | 1.911 | **** |
|  |  | Control FDXR PP1:PP2 vs. Alphas FDXR PP1:PP2 | <0.0001 | 6.604 | **** |
|  |  | Control FDXR PP1:PP2 vs. MB FDXR PP1:PP2 | <0.0001 | 4.538 | **** |
|  |  | X-rays FDXR PP1:PP2 vs. Alphas FDXR PP1:PP2 | 0.0001 | 1.237 | *** |
|  |  | X-rays FDXR PP1:PP2 vs. MB FDXR PP1:PP2 | 0.0007 | 1.014 | *** |
|  |  | Alphas FDXR PP1:PP2 vs. MB FDXR PP1:PP2 | 0.9992 | 0.174 |  |
|  |  | MB FDXR PP1:PP2 vs. EMB FDXR PP1:PP2 | 0.5868 | 0.549 |  |
|  |  |  |  |  |  |
|  | *CDKN1A* | Controls CDKN1A V1:V4 vs. X-rays CDKN1A V1:V4 | >0.9999 | 0.078 |  |
|  |  | Controls CDKN1A V1:V4 vs. Alphas CDKN1A V1:V4 | 0.0007 | 1.250 | *** |
|  |  | Controls CDKN1A V1:V4 vs. MB CDKN1A V1:V4 | 0.0592 | 0.966 |  |
|  |  | X-rays CDKN1A V1:V4 vs. Alphas CDKN1A V1:V4 | 0.0016 | 1.202 | ** |
|  |  | X-rays CDKN1A V1:V4 vs. MB CDKN1A V1:V4 | 0.1047 | 0.907 |  |
|  |  | Alphas CDKN1A V1:V4 vs. MB CDKN1A V1:V4 | 0.7247 | 0.440 |  |
|  |  | MB CDKN1A V1:V4 vs. EMB CDKN1A V1:V4 | >0.9999 | 0.095 |  |
|  |  |  |  |  |  |
|  | *MDM2* | Controls MDM2 315:303/304 vs. X- rays MDM2 315:303/304 | >0.9999 | 0.125 |  |
|  |  | Controls MDM2 315:303/304 vs. Alphas MDM2 315:303/304 | 0.6304 | 0.509 |  |
|  |  | Controls MDM2 315:303/304 vs. MB MDM2 315:303/304 | 0.9039 | 0.380 |  |
|  |  | X- rays MDM2 315:303/304 vs. Alphas MDM2 315:303/304 | 0.8359 | 0.393 |  |
|  |  | X- rays MDM2 315:303/304 vs. MB MDM2 315:303/304 | 0.983 | 0.263 |  |
|  |  | Alphas MDM2 315:303/304 vs. MB MDM2 315:303/304 | 0.9995 | 0.129 |  |
|  |  | MB MDM2 315:303/304 vs. EMB MDM2 315:303/304 | >0.9999 | 0.036 |  |
|  |  |  |  |  |  |
| **4B** | *FDXR* | Control FDXR PP1:PP2 vs. X-rays FDXR PP1:PP2 | <0.0001 | 1.646 | **** |
|  |  | Control FDXR PP1:PP2 vs. Alphas FDXR PP1:PP2 | <0.0001 | 6.145 | **** |
|  |  | Control FDXR PP1:PP2 vs. MB FDXR PP1:PP2 | <0.0001 | 5.341 | **** |
|  |  | X-rays FDXR PP1:PP2 vs. Alphas FDXR PP1:PP2 | 0.0005 | 1.327 | *** |
|  |  | X-rays FDXR PP1:PP2 vs. MB FDXR PP1:PP2 | 0.0026 | 1.154 | ** |
|  |  | Alphas FDXR PP1:PP2 vs. MB FDXR PP1:PP2 | 0.999 | 0.236 |  |
|  |  | MB FDXR PP1:PP2 vs. EMB FDXR PP1:PP2 | 0.7616 | 0.648 |  |
|  |  |  |  |  |  |
|  | *CDKN1A* | Control CDKN1A V1:V4 vs. X-rays CDKN1A V1:V4 | >0.9999 | 0.019 |  |
|  |  | Control CDKN1A V1:V4 vs. Alphas CDKN1A V1:V4 | 0.0051 | 1.277 | ** |
|  |  | Control CDKN1A V1:V4 vs. MB CDKN1A V1:V4 | 0.086 | 1.031 |  |
|  |  | X-rays CDKN1A V1:V4 vs. Alphas CDKN1A V1:V4 | 0.0044 | 1.457 | ** |
|  |  | X-rays CDKN1A V1:V4 vs. MB CDKN1A V1:V4 | 0.0763 | 1.225 |  |
|  |  | Alphas CDKN1A V1:V4 vs. MB CDKN1A V1:V4 | 0.933 | 0.397 |  |
|  |  | MB CDKN1A V1:V4 vs. EMB CDKN1A V1:V4 | 0.9986 | 0.230 |  |
|  |  |  |  |  |  |
|  | *MDM2* | Control MDM2 315:303/304 vs. X-rays MDM2 315:303/304 | >0.9999 | 0.171 |  |
|  |  | Control MDM2 315:303/304 vs. Alphas MDM2 315:303/304 | 0.679 | 0.595 |  |
|  |  | Control MDM2 315:303/304 vs. MB MDM2 315:303/304 | 0.898 | 0.474 |  |
|  |  | X-rays MDM2 315:303/304 vs. Alphas MDM2 315:303/304 | 0.8855 | 0.445 |  |
|  |  | X-rays MDM2 315:303/304 vs. MB MDM2 315:303/304 | 0.9852 | 0.317 |  |
|  |  | Alphas MDM2 315:303/304 vs. MB MDM2 315:303/304 | 0.9999 | 0.131 |  |
|  |  | MB MDM2 315:303/304 vs. EMB MDM2 315:303/304 | >0.9999 | 0.053 |  |
|  |  |  |  |  |  |
| **4C** | *FDXR* | Control FDXR PP1:PP2 vs. X-rays FDXR PP1:PP2 | 0.0016 | 2.483 | ** |
|  |  | Control FDXR PP1:PP2 vs. Alphas FDXR PP1:PP2 | <0.0001 | 7.435 | **** |
|  |  | Control FDXR PP1:PP2 vs. MB FDXR PP1:PP2 | <0.0001 | 3.354 | **** |
|  |  | X-rays FDXR PP1:PP2 vs. Alphas FDXR PP1:PP2 | 0.5208 | 0.983 |  |
|  |  | X-rays FDXR PP1:PP2 vs. MB FDXR PP1:PP2 | 0.5955 | 0.686 |  |
|  |  | Alphas FDXR PP1:PP2 vs. MB FDXR PP1:PP2 | >0.9999 | 0.060 |  |
|  |  | MB FDXR PP1:PP2 vs. EMB FDXR PP1:PP2 | 0.9837 | 0.381 |  |
|  |  |  |  |  |  |
|  | *CDKN1A* | Control CDKN1A V1:V4 vs. X-rays CDKN1A V1:V4 | 0.9997 | 0.291 |  |
|  |  | Control CDKN1A V1:V4 vs. Alphas CDKN1A V1:V4 | 0.3748 | 1.107 |  |
|  |  | Control CDKN1A V1:V4 vs. MB CDKN1A V1:V4 | 0.9349 | 0.757 |  |
|  |  | X-rays CDKN1A V1:V4 vs. Alphas CDKN1A V1:V4 | 0.6478 | 0.779 |  |
|  |  | X-rays CDKN1A V1:V4 vs. MB CDKN1A V1:V4 | 0.9962 | 0.378 |  |
|  |  | Alphas CDKN1A V1:V4 vs. MB CDKN1A V1:V4 | 0.9583 | 0.478 |  |
|  |  | MB CDKN1A V1:V4 vs. EMB CDKN1A V1:V4 | >0.9999 | 0.121 |  |
|  |  |  |  |  |  |
|  | *MDM2* | Control MDM2 315:303/304 vs. X-rays MDM2 315:303/304 | >0.9999 | 0.016 |  |
|  |  | Control MDM2 315:303/304 vs. Alphas MDM2 315:303/304 | 0.3685 | 1.109 |  |
|  |  | Control MDM2 315:303/304 vs. MB MDM2 315:303/304 | 0.9600 | 0.505 |  |
|  |  | X-rays MDM2 315:303/304 vs. Alphas MDM2 315:303/304 | 0.3861 | 1.150 |  |
|  |  | X-rays MDM2 315:303/304 vs. MB MDM2 315:303/304 | 0.9662 | 0.513 |  |
|  |  | Alphas MDM2 315:303/304 vs. MB MDM2 315:303/304 | 0.9291 | 0.594 |  |
|  |  | MB MDM2 315:303/304 vs. EMB MDM2 315:303/304 | >0.9999 | 0.051 |  |
